# 1-(2-Chloro­acet­yl)-3-methyl-2,6-bis­(3,4,5-trimethoxy­phen­yl)piperidine-4-one

**DOI:** 10.1107/S1600536809015864

**Published:** 2009-05-07

**Authors:** B. N. Lakshminarayana, J. Shashidhara Prasad, C. R. Gnanendra, M. A. Sridhar, D. Chenne Gowda

**Affiliations:** a Department of Studies in Physics, Manasagangotri, University of Mysore, Mysore 570 006, India; b Department of Studies in Chemistry, Manasagangotri, University of Mysore, Mysore 570 006, India

## Abstract

In the crystal structure of the title compound, C_26_H_32_ClNO_8_, the piperidine ring is in a twist-chair conformation, with puckering parameters *Q* = 0.655 (4) Å, θ = 93.1 (1) and ϕ = 254.4 (3)°. The *ortho* C atoms of the piperidine ring deviate from the plane defined by the remaining ring atoms by 0.380 (3) and −0.250 (3) Å.

## Related literature

For the biological and pharmacological properties of piperidines, see: Prostakov & Gaivoronskaya (1978 ▶). For the biological activity of piperidones with aryl substituents at the 2 and 6 positions, see: Mobio *et al.* (1989 ▶); Ganellin & Spickett (1965 ▶). For ring-puckering analysis, see: Cremer & Pople (1975 ▶). For the synthesis, see: Baliah *et al.* (1983 ▶).
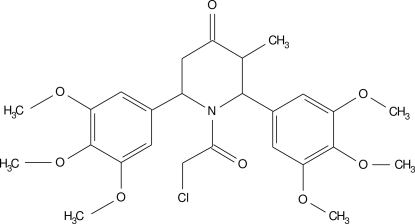


## Experimental

### 

#### Crystal data

C_26_H_32_ClNO_8_
*M* *_r_* = 521.98
Orthorhombic, 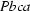

*a* = 13.8720 (8) Å
*b* = 16.5110 (11) Å
*c* = 22.8120 (16) Å
*V* = 5224.9 (6) Å^3^
*Z* = 8
Mo *K*α radiation
μ = 0.20 mm^−1^
*T* = 293 K
0.30 × 0.27 × 0.25 mm

#### Data collection

MacScience DIPLabo 32001 diffractometer
Absorption correction: none
8214 measured reflections
4464 independent reflections
3146 reflections with *I* > 2σ(*I*)
*R*_int_ = 0.027

#### Refinement

*R*[*F* ^2^ > 2σ(*F* ^2^)] = 0.062
*wR*(*F* ^2^) = 0.186
*S* = 1.05
4464 reflections
332 parameters
H-atom parameters constrained
Δρ_max_ = 0.47 e Å^−3^
Δρ_min_ = −0.39 e Å^−3^

### 

Data collection: *XPRESS* (MacScience, 2002 ▶); cell refinement: *SCALEPACK* (Otwinowski & Minor, 1997 ▶); data reduction: *DENZO* (Otwinowski & Minor, 1997 ▶) and *SCALEPACK*; program(s) used to solve structure: *SHELXS97* (Sheldrick, 2008 ▶); program(s) used to refine structure: *SHELXL97* (Sheldrick, 2008 ▶); molecular graphics: *PLATON* (Spek, 2009 ▶) and *ORTEPII* (Johnson, 1976 ▶); software used to prepare material for publication: *PLATON*.

## Supplementary Material

Crystal structure: contains datablocks global, I. DOI: 10.1107/S1600536809015864/nc2143sup1.cif

Structure factors: contains datablocks I. DOI: 10.1107/S1600536809015864/nc2143Isup2.hkl

Additional supplementary materials:  crystallographic information; 3D view; checkCIF report

## Figures and Tables

**Fig. 1.**
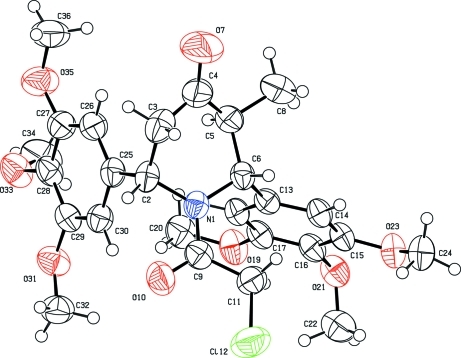
Crystal structure of the title compound with labelling and 50% probability displacement ellipsoids.

## supplementary crystallographic information

### Comment

Piperidines are an important group of compounds in the field of medicinal chemistry owing to the fact that they can frequently be recognized in the structures of numerous naturally occurring alkaloid and synthetic compounds with interesting biological and pharmacological properties (Prostakov  *et al.*, 1978). Furthermore, the significant biological activities of piperridones were associated with aryl substituents at 2 and 6 positions (Ganellin & Spickett, 1965; Mobio *et al.*, 1989). In view of the importance of such compounds the crystal structure of the title compound is reported.

The substituent at C2 is in an equatorial position as indicated by the dihedral angle of 85.18 (2)° between the piperidine and the phenyl ring. The methyl group at C5 reflects C8 and is oriented in  an anti-periplanar conformation as indicated by the torsion angle of N1—C6—C5—C8 = -173.25°. The torsion angle of 180.0 (3)° for C6—N1—C9—O10 shows that O10 is also in an anti-periplanar conformation. The methoxy groups at C27, C25, C17 and C15 are nearly planar with the phenyl ring whereas the methoxy group at C26 and C16 is nearly orthogonal to the phenyl rings.

### Experimental

To a well stirred solution of 3-methyl-2,6-bis(3,4,5-trimethoxyphenyl)piperidine-4-one (Baliah et al., 1983) (5 mmol) and triethylamine(5 mmol) in 30 ml of benzene, chloroacetyl chloride (5 mmol) in 20 ml benzene was added dropwise within about an hour. The resulting mixture was stirred for about 4 hours at room temperature. Afterwards the mixture was quenched in cold water and the organic layer was extracted with ethyl acetate, washed with 5% sodium bicarbonate solution and dried over anhydrous sodium sulphate. Slow evaporation of the solvent leads to crystals of 1-(2-chloroacetyl)-3-methyl-2,6-bis(3,4,5-trimethoxyphenyl) piperidine-4-one.

### Refinement

H atoms were placed at idealized positions and allowed to ride on their parent atoms with C–H distances in the range 0.93–0.98 Å and *U*_iso_(H) = 1.2*U*_eq_(carrier atom; 1.5 for methyl H atoms).

### Crystal data

**Table:**

C_26_H_32_ClNO_8_	*F*(000) = 2208
*M**_r_* = 521.98	*D*_x_ = 1.327 Mg m^−^^3^
Orthorhombic, *P**b**c**a*	Mo *K*α radiation, λ = 0.71073 Å
Hall symbol: -P 2ac 2ab	Cell parameters from 8214 reflections
*a* = 13.8720 (8) Å	θ = 2.9–25.0°
*b* = 16.5110 (11) Å	µ = 0.20 mm^−^^1^
*c* = 22.8120 (16) Å	*T* = 293 K
*V* = 5224.9 (6) Å^3^	Block, white
*Z* = 8	0.30 × 0.27 × 0.25 mm

### Data collection

**Table:**

MacScience DIPLabo 32001 diffractometer	3146 reflections with *I* > 2σ(*I*)
Radiation source: fine-focus sealed tube	*R*_int_ = 0.027
graphite	θ_max_ = 25.0°, θ_min_ = 2.9°
Detector resolution: 10.0 pixels mm^-1^	*h* = −16→16
ω scans	*k* = −19→19
8214 measured reflections	*l* = −27→26
4464 independent reflections	

### Refinement

**Table:**

Refinement on *F*^2^	Primary atom site location: structure-invariant direct methods
Least-squares matrix: full	Secondary atom site location: difference Fourier map
*R*[*F*^2^ > 2σ(*F*^2^)] = 0.062	Hydrogen site location: inferred from neighbouring sites
*wR*(*F*^2^) = 0.186	H-atom parameters constrained
*S* = 1.05	*w* = 1/[σ^2^(*F*_o_^2^) + (0.0931*P*)^2^ + 2.926*P*] where *P* = (*F*_o_^2^ + 2*F*_c_^2^)/3
4464 reflections	(Δ/σ)_max_ < 0.001
332 parameters	Δρ_max_ = 0.47 e Å^−^^3^
0 restraints	Δρ_min_ = −0.39 e Å^−^^3^

### Special details

**Table:**

Geometry. Bond distances, angles *etc*. have been calculated using the rounded fractional coordinates. All su's are estimated from the variances of the (full) variance-covariance matrix. The cell e.s.d.'s are taken into account in the estimation of distances, angles and torsion angles
Refinement. Refinement of *F*^2^ against ALL reflections. The weighted *R*-factor *wR* and goodness of fit *S* are based on *F*^2^, conventional *R*-factors *R* are based on *F*, with *F* set to zero for negative *F*^2^. The threshold expression of *F*^2^ > σ(*F*^2^) is used only for calculating *R*-factors(gt) *etc*. and is not relevant to the choice of reflections for refinement. *R*-factors based on *F*^2^ are statistically about twice as large as those based on *F*, and *R*- factors based on ALL data will be even larger.

### Fractional atomic coordinates and isotropic or equivalent isotropic displacement parameters (Å^2^)

**Table:**

	*x*	*y*	*z*	*U*_iso_*/*U*_eq_	
Cl12	0.14569 (9)	−0.00580 (6)	0.36264 (6)	0.1014 (5)	
O7	0.0245 (3)	−0.4484 (3)	0.28056 (17)	0.1467 (19)	
O10	0.07651 (19)	−0.14395 (16)	0.42507 (10)	0.0742 (9)	
O19	0.49642 (15)	−0.28235 (15)	0.40351 (9)	0.0623 (8)	
O21	0.57332 (13)	−0.19912 (15)	0.31391 (9)	0.0584 (8)	
O23	0.46432 (15)	−0.15115 (15)	0.22323 (9)	0.0595 (8)	
O31	0.27178 (17)	−0.31372 (15)	0.56956 (9)	0.0649 (8)	
O33	0.26328 (19)	−0.47563 (15)	0.57353 (9)	0.0725 (9)	
O35	0.1596 (2)	−0.55902 (15)	0.49519 (11)	0.0847 (10)	
N1	0.12281 (16)	−0.24570 (15)	0.36489 (10)	0.0473 (8)	
C2	0.0590 (2)	−0.3025 (2)	0.39713 (13)	0.0524 (10)	
C3	−0.0007 (3)	−0.3497 (2)	0.35294 (16)	0.0690 (14)	
C4	0.0569 (3)	−0.3931 (2)	0.30853 (16)	0.0760 (16)	
C5	0.1608 (3)	−0.3656 (2)	0.30122 (14)	0.0603 (11)	
C6	0.1732 (2)	−0.27421 (18)	0.31092 (12)	0.0476 (10)	
C8	0.2024 (4)	−0.3910 (3)	0.24254 (19)	0.0953 (19)	
C9	0.1181 (2)	−0.1667 (2)	0.38063 (14)	0.0537 (11)	
C11	0.1658 (3)	−0.10579 (19)	0.34041 (16)	0.0613 (12)	
C13	0.2804 (2)	−0.25428 (18)	0.31204 (12)	0.0447 (9)	
C14	0.3213 (2)	−0.21277 (19)	0.26566 (12)	0.0488 (10)	
C15	0.4183 (2)	−0.19340 (19)	0.26646 (12)	0.0485 (10)	
C16	0.4755 (2)	−0.21760 (19)	0.31333 (13)	0.0497 (10)	
C17	0.4349 (2)	−0.26141 (19)	0.35897 (12)	0.0482 (10)	
C18	0.3374 (2)	−0.27934 (18)	0.35895 (12)	0.0466 (9)	
C20	0.4600 (3)	−0.3312 (2)	0.44945 (14)	0.0661 (11)	
C22	0.5942 (2)	−0.1200 (2)	0.33503 (18)	0.0742 (14)	
C24	0.4061 (3)	−0.1076 (2)	0.18305 (15)	0.0661 (11)	
C25	0.1140 (2)	−0.3524 (2)	0.44265 (13)	0.0507 (10)	
C26	0.1100 (2)	−0.4354 (2)	0.44530 (14)	0.0576 (11)	
C27	0.1605 (3)	−0.4770 (2)	0.48873 (14)	0.0593 (11)	
C28	0.2159 (2)	−0.4349 (2)	0.52945 (12)	0.0551 (11)	
C29	0.2184 (2)	−0.3514 (2)	0.52730 (12)	0.0520 (10)	
C30	0.1675 (2)	−0.3100 (2)	0.48416 (13)	0.0521 (10)	
C32	0.2524 (3)	−0.2308 (2)	0.58099 (17)	0.0713 (12)	
C34	0.3539 (3)	−0.5089 (3)	0.55613 (19)	0.0890 (17)	
C36	0.1045 (4)	−0.6052 (2)	0.4561 (2)	0.0960 (18)	
H2	0.01350	−0.26880	0.41910	0.0630*	
H3A	−0.04010	−0.38860	0.37390	0.0830*	
H3B	−0.04380	−0.31240	0.33310	0.0830*	
H5	0.19860	−0.39300	0.33160	0.0720*	
H6	0.14450	−0.24620	0.27730	0.0570*	
H8A	0.18970	−0.44750	0.23610	0.1420*	
H8B	0.27080	−0.38190	0.24250	0.1420*	
H8C	0.17310	−0.35970	0.21190	0.1420*	
H11A	0.14120	−0.11290	0.30100	0.0740*	
H11B	0.23470	−0.11600	0.33950	0.0740*	
H14	0.28350	−0.19780	0.23380	0.0590*	
H18	0.31020	−0.30790	0.39000	0.0560*	
H20A	0.40750	−0.30360	0.46830	0.0990*	
H20B	0.51010	−0.34130	0.47750	0.0990*	
H20C	0.43750	−0.38170	0.43370	0.0990*	
H22A	0.56350	−0.08060	0.31040	0.1120*	
H22B	0.66270	−0.11150	0.33460	0.1120*	
H22C	0.57060	−0.11460	0.37440	0.1120*	
H24A	0.37270	−0.14500	0.15800	0.0990*	
H24B	0.44600	−0.07280	0.15970	0.0990*	
H24C	0.36010	−0.07550	0.20420	0.0990*	
H26	0.07340	−0.46390	0.41810	0.0690*	
H30	0.16930	−0.25370	0.48300	0.0630*	
H32A	0.27190	−0.19880	0.54790	0.1070*	
H32B	0.28760	−0.21390	0.61510	0.1070*	
H32C	0.18460	−0.22360	0.58770	0.1070*	
H34A	0.34540	−0.54120	0.52150	0.1340*	
H34B	0.37900	−0.54200	0.58710	0.1340*	
H34C	0.39830	−0.46570	0.54800	0.1340*	
H36A	0.03760	−0.59130	0.46030	0.1440*	
H36B	0.11310	−0.66170	0.46460	0.1440*	
H36C	0.12490	−0.59440	0.41670	0.1440*	

### Atomic displacement parameters (Å^2^)

**Table:**

	*U*^11^	*U*^22^	*U*^33^	*U*^12^	*U*^13^	*U*^23^
Cl12	0.1112 (9)	0.0590 (6)	0.1341 (10)	−0.0100 (6)	0.0317 (8)	−0.0153 (6)
O7	0.159 (3)	0.153 (4)	0.128 (3)	−0.093 (3)	0.024 (3)	−0.062 (3)
O10	0.0898 (18)	0.0728 (17)	0.0599 (14)	0.0103 (14)	0.0180 (14)	0.0001 (12)
O19	0.0498 (12)	0.0887 (18)	0.0483 (12)	0.0076 (11)	−0.0098 (10)	0.0087 (11)
O21	0.0394 (11)	0.0803 (16)	0.0554 (12)	0.0056 (10)	0.0030 (10)	−0.0077 (11)
O23	0.0507 (12)	0.0819 (16)	0.0460 (11)	0.0024 (11)	0.0044 (10)	0.0106 (11)
O31	0.0698 (14)	0.0686 (15)	0.0563 (13)	0.0078 (12)	−0.0193 (12)	−0.0053 (11)
O33	0.0930 (18)	0.0727 (16)	0.0519 (12)	0.0264 (14)	−0.0143 (12)	0.0041 (11)
O35	0.123 (2)	0.0532 (15)	0.0780 (17)	0.0040 (14)	−0.0279 (16)	0.0027 (13)
N1	0.0439 (13)	0.0544 (15)	0.0437 (13)	−0.0017 (11)	−0.0038 (11)	0.0064 (11)
C2	0.0450 (16)	0.065 (2)	0.0471 (17)	−0.0039 (14)	−0.0004 (14)	0.0133 (14)
C3	0.0561 (19)	0.082 (3)	0.069 (2)	−0.0179 (18)	−0.0134 (18)	0.0181 (19)
C4	0.091 (3)	0.078 (3)	0.059 (2)	−0.032 (2)	−0.010 (2)	−0.0022 (19)
C5	0.074 (2)	0.059 (2)	0.0480 (17)	−0.0071 (16)	−0.0056 (16)	−0.0033 (15)
C6	0.0459 (16)	0.0573 (19)	0.0395 (14)	−0.0012 (13)	−0.0030 (13)	0.0037 (13)
C8	0.122 (4)	0.084 (3)	0.080 (3)	−0.014 (3)	0.013 (3)	−0.028 (2)
C9	0.0534 (18)	0.059 (2)	0.0488 (17)	0.0052 (14)	−0.0093 (15)	0.0025 (15)
C11	0.063 (2)	0.053 (2)	0.068 (2)	−0.0030 (15)	0.0041 (17)	0.0006 (16)
C13	0.0458 (15)	0.0477 (16)	0.0405 (14)	0.0050 (13)	−0.0035 (13)	−0.0027 (12)
C14	0.0482 (16)	0.0592 (19)	0.0391 (15)	0.0065 (13)	−0.0035 (13)	0.0010 (13)
C15	0.0464 (16)	0.0610 (19)	0.0382 (15)	0.0056 (14)	0.0041 (13)	−0.0047 (13)
C16	0.0434 (16)	0.0617 (19)	0.0440 (16)	0.0083 (13)	0.0020 (13)	−0.0080 (14)
C17	0.0460 (16)	0.0606 (19)	0.0381 (15)	0.0123 (14)	−0.0033 (13)	−0.0044 (13)
C18	0.0491 (16)	0.0526 (18)	0.0382 (14)	0.0045 (13)	−0.0007 (13)	0.0010 (13)
C20	0.074 (2)	0.072 (2)	0.0524 (19)	0.0114 (18)	−0.0165 (18)	0.0106 (17)
C22	0.0535 (19)	0.091 (3)	0.078 (2)	−0.0043 (19)	0.0008 (19)	−0.019 (2)
C24	0.065 (2)	0.078 (2)	0.0553 (19)	0.0018 (18)	0.0036 (17)	0.0149 (17)
C25	0.0483 (16)	0.060 (2)	0.0438 (16)	−0.0018 (14)	−0.0009 (14)	0.0072 (14)
C26	0.066 (2)	0.058 (2)	0.0489 (17)	−0.0037 (16)	−0.0071 (16)	0.0021 (15)
C27	0.075 (2)	0.0498 (19)	0.0531 (18)	0.0052 (16)	−0.0015 (17)	0.0044 (15)
C28	0.066 (2)	0.059 (2)	0.0404 (15)	0.0137 (16)	−0.0069 (15)	0.0030 (14)
C29	0.0525 (17)	0.061 (2)	0.0424 (16)	0.0044 (14)	−0.0017 (14)	−0.0024 (14)
C30	0.0540 (17)	0.0533 (18)	0.0491 (16)	0.0016 (14)	−0.0026 (15)	0.0063 (14)
C32	0.080 (2)	0.064 (2)	0.070 (2)	−0.0091 (18)	−0.011 (2)	−0.0067 (18)
C34	0.096 (3)	0.085 (3)	0.086 (3)	0.038 (2)	−0.026 (2)	−0.012 (2)
C36	0.147 (4)	0.056 (2)	0.085 (3)	−0.007 (3)	−0.022 (3)	−0.010 (2)

### Geometric parameters (Å, °)

**Table:**

Cl12—C11	1.749 (3)	C27—C28	1.392 (5)
O7—C4	1.201 (6)	C28—C29	1.380 (5)
O10—C9	1.225 (4)	C29—C30	1.391 (4)
O19—C17	1.371 (3)	C2—H2	0.9800
O19—C20	1.416 (4)	C3—H3A	0.9700
O21—C16	1.391 (3)	C3—H3B	0.9700
O21—C22	1.422 (4)	C5—H5	0.9800
O23—C15	1.366 (4)	C6—H6	0.9800
O23—C24	1.418 (4)	C8—H8A	0.9600
O31—C29	1.366 (4)	C8—H8B	0.9600
O31—C32	1.419 (4)	C8—H8C	0.9600
O33—C28	1.377 (4)	C11—H11A	0.9700
O33—C34	1.428 (5)	C11—H11B	0.9700
O35—C27	1.362 (4)	C14—H14	0.9300
O35—C36	1.400 (5)	C18—H18	0.9300
N1—C2	1.485 (4)	C20—H20A	0.9600
N1—C6	1.492 (4)	C20—H20B	0.9600
N1—C9	1.355 (4)	C20—H20C	0.9600
C2—C3	1.520 (5)	C22—H22A	0.9600
C2—C25	1.529 (4)	C22—H22B	0.9600
C3—C4	1.476 (5)	C22—H22C	0.9600
C4—C5	1.520 (6)	C24—H24A	0.9600
C5—C6	1.535 (4)	C24—H24B	0.9600
C5—C8	1.517 (6)	C24—H24C	0.9600
C6—C13	1.523 (4)	C26—H26	0.9300
C9—C11	1.514 (5)	C30—H30	0.9300
C13—C14	1.382 (4)	C32—H32A	0.9600
C13—C18	1.393 (4)	C32—H32B	0.9600
C14—C15	1.383 (4)	C32—H32C	0.9600
C15—C16	1.390 (4)	C34—H34A	0.9600
C16—C17	1.387 (4)	C34—H34B	0.9600
C17—C18	1.385 (4)	C34—H34C	0.9600
C25—C26	1.373 (5)	C36—H36A	0.9600
C25—C30	1.392 (4)	C36—H36B	0.9600
C26—C27	1.394 (5)	C36—H36C	0.9600
			
C17—O19—C20	118.0 (3)	C4—C5—H5	107.00
C16—O21—C22	113.8 (2)	C6—C5—H5	107.00
C15—O23—C24	117.4 (2)	C8—C5—H5	107.00
C29—O31—C32	117.8 (3)	N1—C6—H6	108.00
C28—O33—C34	113.9 (3)	C5—C6—H6	108.00
C27—O35—C36	118.5 (3)	C13—C6—H6	108.00
C2—N1—C6	119.3 (2)	C5—C8—H8A	109.00
C2—N1—C9	116.6 (2)	C5—C8—H8B	109.00
C6—N1—C9	123.0 (2)	C5—C8—H8C	109.00
N1—C2—C3	108.7 (2)	H8A—C8—H8B	109.00
N1—C2—C25	112.3 (2)	H8A—C8—H8C	110.00
C3—C2—C25	116.5 (3)	H8B—C8—H8C	109.00
C2—C3—C4	114.2 (3)	Cl12—C11—H11A	109.00
O7—C4—C3	122.1 (4)	Cl12—C11—H11B	109.00
O7—C4—C5	121.6 (4)	C9—C11—H11A	109.00
C3—C4—C5	116.3 (3)	C9—C11—H11B	109.00
C4—C5—C6	112.6 (3)	H11A—C11—H11B	108.00
C4—C5—C8	112.0 (3)	C13—C14—H14	120.00
C6—C5—C8	110.9 (3)	C15—C14—H14	120.00
N1—C6—C5	112.1 (2)	C13—C18—H18	120.00
N1—C6—C13	112.1 (2)	C17—C18—H18	120.00
C5—C6—C13	108.9 (3)	O19—C20—H20A	109.00
O10—C9—N1	122.5 (3)	O19—C20—H20B	110.00
O10—C9—C11	120.2 (3)	O19—C20—H20C	109.00
N1—C9—C11	117.3 (3)	H20A—C20—H20B	109.00
Cl12—C11—C9	112.4 (3)	H20A—C20—H20C	109.00
C6—C13—C14	119.7 (2)	H20B—C20—H20C	110.00
C6—C13—C18	120.2 (2)	O21—C22—H22A	110.00
C14—C13—C18	120.1 (3)	O21—C22—H22B	109.00
C13—C14—C15	120.3 (3)	O21—C22—H22C	109.00
O23—C15—C14	124.3 (3)	H22A—C22—H22B	110.00
O23—C15—C16	115.8 (2)	H22A—C22—H22C	109.00
C14—C15—C16	119.9 (3)	H22B—C22—H22C	109.00
O21—C16—C15	120.1 (3)	O23—C24—H24A	109.00
O21—C16—C17	120.2 (3)	O23—C24—H24B	110.00
C15—C16—C17	119.7 (3)	O23—C24—H24C	110.00
O19—C17—C16	115.8 (2)	H24A—C24—H24B	109.00
O19—C17—C18	123.7 (3)	H24A—C24—H24C	109.00
C16—C17—C18	120.5 (3)	H24B—C24—H24C	109.00
C13—C18—C17	119.4 (3)	C25—C26—H26	120.00
C2—C25—C26	123.2 (3)	C27—C26—H26	120.00
C2—C25—C30	117.2 (3)	C25—C30—H30	120.00
C26—C25—C30	119.6 (3)	C29—C30—H30	120.00
C25—C26—C27	120.2 (3)	O31—C32—H32A	109.00
O35—C27—C26	124.2 (3)	O31—C32—H32B	109.00
O35—C27—C28	115.4 (3)	O31—C32—H32C	110.00
C26—C27—C28	120.4 (3)	H32A—C32—H32B	110.00
O33—C28—C27	120.5 (3)	H32A—C32—H32C	109.00
O33—C28—C29	120.1 (3)	H32B—C32—H32C	109.00
C27—C28—C29	119.3 (3)	O33—C34—H34A	110.00
O31—C29—C28	116.3 (3)	O33—C34—H34B	110.00
O31—C29—C30	123.4 (3)	O33—C34—H34C	109.00
C28—C29—C30	120.2 (3)	H34A—C34—H34B	110.00
C25—C30—C29	120.3 (3)	H34A—C34—H34C	109.00
N1—C2—H2	106.00	H34B—C34—H34C	109.00
C3—C2—H2	106.00	O35—C36—H36A	109.00
C25—C2—H2	106.00	O35—C36—H36B	109.00
C2—C3—H3A	109.00	O35—C36—H36C	110.00
C2—C3—H3B	109.00	H36A—C36—H36B	109.00
C4—C3—H3A	109.00	H36A—C36—H36C	110.00
C4—C3—H3B	109.00	H36B—C36—H36C	109.00
H3A—C3—H3B	108.00		
			
C20—O19—C17—C18	5.9 (4)	C4—C5—C6—C13	171.4 (3)
C20—O19—C17—C16	−176.3 (3)	C5—C6—C13—C18	−69.9 (3)
C22—O21—C16—C17	−97.6 (3)	N1—C6—C13—C18	54.7 (4)
C22—O21—C16—C15	83.5 (4)	N1—C6—C13—C14	−126.7 (3)
C24—O23—C15—C14	16.4 (4)	C5—C6—C13—C14	108.6 (3)
C24—O23—C15—C16	−163.9 (3)	N1—C9—C11—Cl12	175.0 (2)
C32—O31—C29—C30	19.2 (4)	O10—C9—C11—Cl12	−4.3 (4)
C32—O31—C29—C28	−160.1 (3)	C6—C13—C18—C17	179.4 (3)
C34—O33—C28—C29	−100.9 (4)	C18—C13—C14—C15	−2.3 (5)
C34—O33—C28—C27	82.9 (4)	C14—C13—C18—C17	0.8 (4)
C36—O35—C27—C28	179.3 (3)	C6—C13—C14—C15	179.2 (3)
C36—O35—C27—C26	−0.2 (5)	C13—C14—C15—C16	1.7 (5)
C9—N1—C2—C25	−103.5 (3)	C13—C14—C15—O23	−178.6 (3)
C6—N1—C9—C11	0.7 (4)	O23—C15—C16—O21	−0.6 (4)
C2—N1—C6—C5	−7.8 (3)	O23—C15—C16—C17	−179.5 (3)
C2—N1—C9—O10	11.9 (4)	C14—C15—C16—O21	179.2 (3)
C9—N1—C2—C3	126.2 (3)	C14—C15—C16—C17	0.3 (5)
C6—N1—C2—C3	−42.4 (3)	C15—C16—C17—O19	−179.6 (3)
C2—N1—C9—C11	−167.4 (3)	C15—C16—C17—C18	−1.7 (5)
C6—N1—C9—O10	−180.0 (3)	O21—C16—C17—C18	179.4 (3)
C9—N1—C6—C5	−175.6 (3)	O21—C16—C17—O19	1.5 (4)
C6—N1—C2—C25	88.0 (3)	O19—C17—C18—C13	178.9 (3)
C9—N1—C6—C13	61.6 (3)	C16—C17—C18—C13	1.2 (4)
C2—N1—C6—C13	−130.7 (3)	C2—C25—C30—C29	179.2 (3)
C3—C2—C25—C26	−1.1 (4)	C30—C25—C26—C27	−1.0 (5)
C3—C2—C25—C30	−178.8 (3)	C26—C25—C30—C29	1.4 (4)
N1—C2—C3—C4	55.7 (4)	C2—C25—C26—C27	−178.7 (3)
N1—C2—C25—C26	−127.3 (3)	C25—C26—C27—C28	−0.5 (5)
C25—C2—C3—C4	−72.3 (4)	C25—C26—C27—O35	179.0 (3)
N1—C2—C25—C30	55.0 (3)	O35—C27—C28—O33	−1.7 (5)
C2—C3—C4—C5	−17.8 (4)	O35—C27—C28—C29	−177.9 (3)
C2—C3—C4—O7	160.5 (4)	C26—C27—C28—C29	1.6 (5)
C3—C4—C5—C8	−159.8 (3)	C26—C27—C28—O33	177.9 (3)
O7—C4—C5—C6	147.7 (4)	O33—C28—C29—O31	1.8 (4)
C3—C4—C5—C6	−34.0 (4)	C27—C28—C29—C30	−1.2 (4)
O7—C4—C5—C8	21.9 (5)	O33—C28—C29—C30	−177.5 (3)
C8—C5—C6—C13	−62.2 (4)	C27—C28—C29—O31	178.1 (3)
C8—C5—C6—N1	173.2 (3)	O31—C29—C30—C25	−179.6 (3)
C4—C5—C6—N1	46.8 (3)	C28—C29—C30—C25	−0.3 (4)
